# Tumoral Melanosis: A Benign Mimicker of Malignant Disease

**DOI:** 10.7759/cureus.99407

**Published:** 2025-12-16

**Authors:** Analy Gomez-Picos, Carol E Marquez Maldonado, Dolores M Arellano Vivero, Lucia Achell Nava, Maria A Loredo Alanis

**Affiliations:** 1 Dermatology, Tecnologico de Monterrey, Escuela de Medicina y Ciencias de la Salud, Monterrey, MEX; 2 Dermatology, Centro Médico Nacional (CMN) “20 de Noviembre”, Instituto de Seguridad y Servicios Sociales de los Trabajadores del Estado (ISSSTE), Mexico City, MEX; 3 Faculty of Health Sciences, Centro Médico Nacional (CMN) “20 de Noviembre”, Mexico City, MEX; 4 Pathology, Centro Médico Nacional (CMN) “20 de Noviembre”, Instituto de Seguridad y Servicios Sociales de los Trabajadores del Estado (ISSSTE), Mexico City, MEX

**Keywords:** immunotherapy response, malignant melanoma metastasis, melanoma and immunotherapy, melanoma diagnosis, tumoral melanosis

## Abstract

We report the case of a 60-year-old male with metastatic melanoma originating from a hyperpigmented lesion on the hard palate. During treatment with nivolumab, ipilimumab, cisplatin, and temozolomide, he developed multiple bluish macule-like lesions on the retroauricular region, neck, and anterior chest, surrounded by vitiligo-like halos. Given the clinical suspicion of in-transit metastases, biopsies were performed. Histopathological evaluation revealed dermal aggregates of melanin-laden macrophages, positive for CD68, in the absence of melanocytes, findings characteristic of tumoral melanosis. This case underscores the importance of biopsy for new pigmented lesions arising during melanoma immunotherapy. Recognizing tumoral melanosis is essential to avoid misinterpretation as disease progression and to guide appropriate clinical management.

## Introduction

Melanoma accounts for 90% of skin cancer-related mortality, with the metastatic form occurring in approximately 10%-15% of cases [1]. In these patients, immunotherapy has been shown to improve prognosis [2]. Tumoral melanosis (TM) is an uncommon dermatologic manifestation characterized by dense dermal aggregates of melanin-laden macrophages without viable melanoma cells, typically reflecting a regressive process. Although benign, TM can clinically mimic primary or metastatic melanoma. This entity has become increasingly recognized in patients treated with immune checkpoint inhibitors, yet its prognostic implications remain uncertain, with available evidence describing mixed clinical outcomes [3]. We present this case to highlight the importance of accurate recognition of TM in the era of immunotherapy and underscore the need for histopathologic confirmation when new pigmented lesions arise in patients treated for melanoma.

## Case presentation

**Figure 1:**
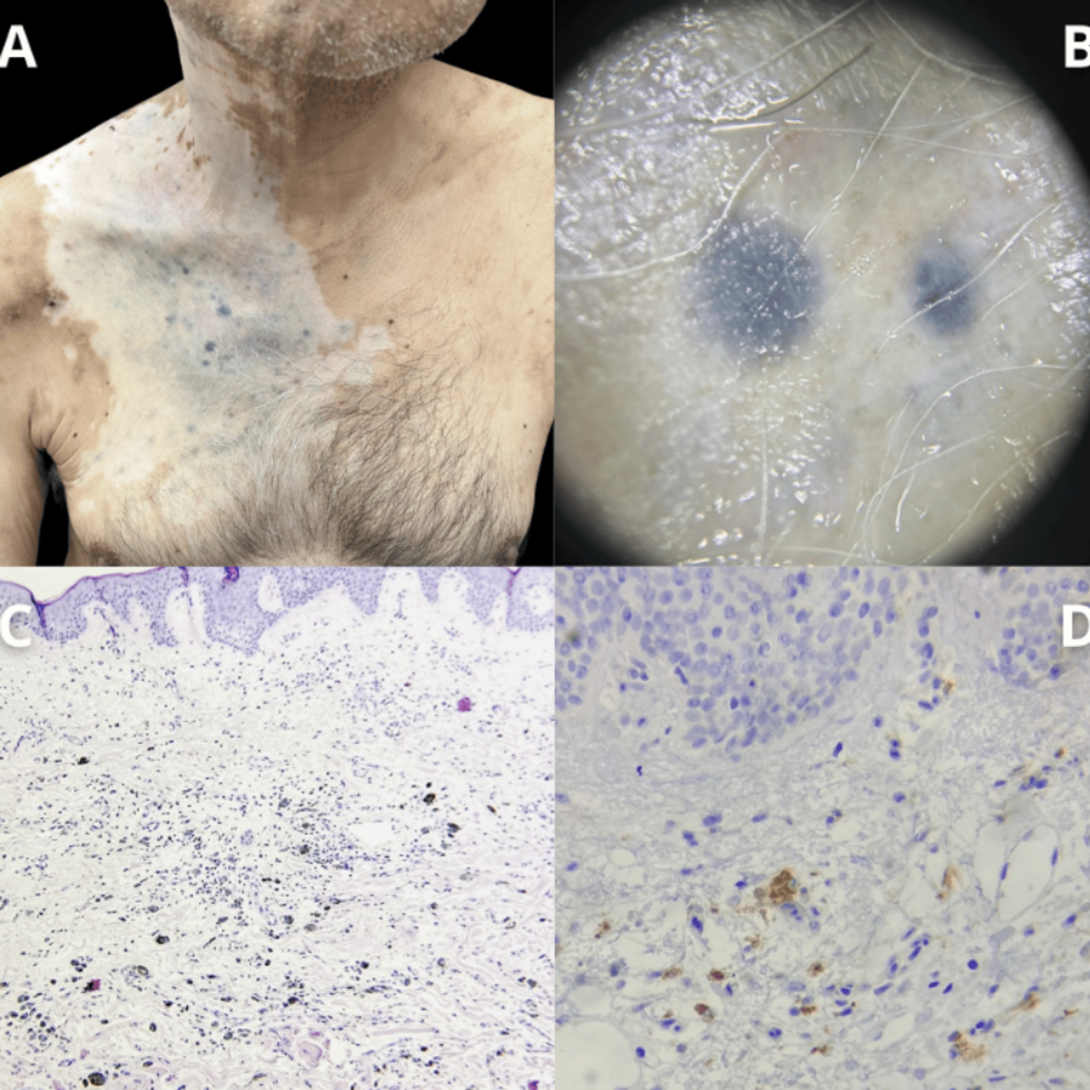
Clinical and histopathological findings. (A) Bluish macule-like neoplasms of varying sizes with well-defined borders, surrounded by a vitiligo-like perilesional background. (B) Dermoscopy of the lesions. (C) Panoramic histologic findings showing dermal macrophages laden with melanin. (D) Immunohistochemistry demonstrating CD68-positive dermal macrophages.

A 60-year-old male with a one-year history of metastatic melanoma to the neck, initially presenting as a hyperpigmented tumor on the hard palate, was undergoing treatment with nivolumab, ipilimumab, cisplatin, and temozolomide. Eight months later presented disseminated lesions affecting the right retroauricular region, neck, and anterior chest, characterized by bluish macule-like neoplasms of varying sizes with well-defined borders, surrounded by a vitiligo-like perilesional background. Biopsies of two lesions were performed due to suspicion of in-transit metastases. Histopathological analysis revealed dermal macrophages laden with melanin (CD68-positive on immunohistochemistry), but no melanocytes were present (Figure 1).

## Discussion

TM can clinically mimic metastatic melanoma and represents an uncommon manifestation of tumoral regression. It should be suspected in patients receiving treatment with monoclonal antibodies targeting PD-1 (nivolumab, pembrolizumab) or CTLA-4 inhibitors (ipilimumab). TM may present as bluish macules, papules, or nodules that are clinically indistinguishable from primary or metastatic melanoma [4]. A retrospective case series of patients with metastatic melanoma published in 2024 described a prevalence of 7.4% of at least one lesion of TM, and these were clinical and histopathologically consistent with what is described in published literature [3]. Differential diagnosis includes melanocytic lesions such as blue nevus and pigmented epithelioid melanocytoma [5]. Diagnostic confirmation requires histopathological correlation through immunohistochemistry, demonstrating the absence of melanocytes and the presence of dermal CD68+ melanin-laden macrophages, resulting from the phagocytosis of melanin released during melanoma cell degradation. The prognostic significance of these findings is yet to be established, as the available evidence reports conflicting outcomes. A case series of 10 patients who developed TM after immunotherapy described disease progression in 10% of the subjects despite having developed TM lesions; nonetheless, 80% showed a complete or partial response [6].

## Conclusions

TM remains a rare and often underrecognized manifestation of melanoma regression, making its diagnosis challenging and its prognostic implications uncertain. The lack of published cases perpetuates the difficulty in distinguishing TM from active metastatic disease based solely on clinical appearance, especially in patients undergoing immune checkpoint inhibitor therapy. This case highlights the importance of timely biopsy of suspicious lesions in melanoma patients undergoing immunotherapy and emphasizes the need for continued reporting to improve understanding of TM clinical relevance.

## References

[1] Garbe C, Amaral T, Peris K (2022). European consensus-based interdisciplinary guideline for melanoma. Part 1: Diagnostics: Update 2022. Eur J Cancer.

[2] Ralli M, Botticelli A, Visconti IC (2020). Immunotherapy in the treatment of metastatic melanoma: current knowledge and future directions. J Immunol Res.

[3] Wix SN, Heberton M, Vandergriff TW, Yancey KB, Gill JG (2024). Tumoral melanosis: a case series of patients with metastatic melanoma after systemic immunotherapy. JAAD Case Rep.

[4] Jurgens A, Guru S, Guo R, Brewer J, Bridges A, Jakub J, Comfere N (2021). Tumoral melanosis in the setting of targeted immunotherapy for metastatic melanoma: a single institutional experience and literature review. Am J Dermatopathol.

[5] Potter AJ, Ferguson PM, Lo SN (2024). The prognostic significance of tumoral melanosis. J Cutan Pathol.

[6] Arias NM, Peñaranda JM, Gaspar LS, Mosquera AC, Sánchez-Aguilar D (2022). Tumoral melanosis associated with nivolumab therapy for metastatic melanoma. J Dtsch Dermatol Ges.

